# Heat stress in spermatogenesis: a redox‐dependent decision network linking ROS, autophagy, and regulated cell death

**DOI:** 10.1002/brv.70200

**Published:** 2026-07-10

**Authors:** Ribrio Ivan Tavares Pereira Batista

**Affiliations:** ^1^ Instituto de Biodiversidade e Sustentabilidade (NUPEM) Universidade Federal do Rio de Janeiro (UFRJ) Av. Amaro Reinaldo dos Santos Silva, 764, São José do Barreto Macaé RJ 27965‐045 Brazil; ^2^ Programa de Pós‐Graduação em Ciência e Biotecnologia (PPBI) Universidade Federal Fluminense Rua Professor Marcos Waldemar de Freitas Reis, Bloco M, sala 229C São Domingos Niterói CEP 24210‐201 RJ Brazil

**Keywords:** oxidative stress, redox signalling, ferroptosis, pyroptosis, mitochondrial dysfunction, Sertoli cells, male fertility, lipid peroxidation

## Abstract

Heat stress (HS) is an increasing threat to male reproductive health, disrupting spermatogenesis through complex and interdependent cellular responses within the seminiferous epithelium. Although excessive reactive oxygen species (ROS) production is a hallmark of HS, ROS also act as central regulators of cell fate decisions beyond their role in oxidative damage. Here, we propose a unified framework in which spermatogenic outcomes under HS are governed by a redox‐dependent decision network, whereby ROS dynamics determine the balance between adaptive responses and regulated cell death (RCD). At moderate levels, ROS activate cytoprotective mechanisms, including antioxidant defences and autophagy, thereby promoting proteostasis and preserving cellular homeostasis. In contrast, excessive or sustained ROS accumulation induces lipid peroxidation, mitochondrial dysfunction, and impaired autophagic flux, driving the transition toward interconnected RCD pathways, including apoptosis, ferroptosis, and pyroptosis. Autophagy emerges as a central regulatory checkpoint in this network, acting as a molecular rheostat that modulates the shift between adaptation and cell death. Importantly, HS effects are stage‐specific across spermatogenesis. Spermatogonia exhibit relative resilience due to efficient redox buffering and autophagic competence, whereas spermatocytes represent a critical vulnerability point, marked by heightened oxidative sensitivity and a strong propensity toward apoptosis. Post‐meiotic germ cells are particularly susceptible to disruptions in structural remodelling and membrane integrity, while Sertoli cells act as key regulators of the redox microenvironment and amplifiers of HS‐induced damage. Finally, we highlight redox balance modulation as a promising strategy to mitigate HS‐induced reproductive damage. However, effective antioxidant interventions must be precisely calibrated to preserve physiological redox signalling and autophagic function rather than indiscriminately suppress ROS. Together, this framework provides a mechanistic basis for understanding how HS regulates germ cell fate and identifies potential targets for preserving male fertility under environmental and physiological thermal stress.

## I. INTRODUCTION

A decade after the Paris Agreement established the goal of limiting global warming to 1.5 °C above pre‐industrial levels, the world is already experiencing unprecedented thermal stress, with global temperatures exceeding this threshold in recent years (Ebi *et al*., 2021; Romanello *et al*., 2022). The increasing frequency, intensity, and duration of heatwaves pose a major threat to biological systems, with far‐reaching consequences for biodiversity, human health, and food security. Among the physiological systems affected by rising temperatures, male reproduction is particularly vulnerable. Spermatogenesis depends on a tightly regulated thermal environment and operates within a narrow physiological temperature range, making the testis highly susceptible to thermal perturbations (Becker *et al*., 2020; Robinson *et al*., 2023). Yet, despite decades of research documenting the detrimental effects of heat stress (HS) on male fertility, current models largely focus on individual molecular pathways and fail to explain how germ cells integrate stress signals to determine whether they adapt, survive, or undergo regulated cell death.

In mammals, spermatogenesis occurs within the seminiferous epithelium at temperatures approximately 2–4 °C below core body temperature, a gradient maintained by specialized anatomical and physiological mechanisms, including scrotal thermoregulation and counter‐current heat exchange (Mieusset & Bujan, 1995; Setchell, 1998; Durairajanayagam, Agarwal & Ong, 2015). Disruption of this thermal balance elevates testicular temperature and triggers a cascade of cellular responses that ultimately compromise germ cell development and fertility (Kastelic, Rizzoto & Thundathil, 2018; Abdelnour *et al*., 2019). However, the biological consequences of HS are not uniform. Instead, they depend on the intensity, duration, and frequency of thermal exposure, generating outcomes that range from successful cellular adaptation to irreversible tissue damage and reproductive failure.

We propose that these divergent outcomes are not determined solely by the magnitude of cellular injury, but by the ability of germ cells to sense, integrate, and respond to alterations in redox homeostasis. In this perspective, HS acts as a regulator of a redox‐dependent cell fate decision network, in which cellular responses emerge from the dynamic interplay between oxidative signalling, stress adaptation mechanisms, and regulated cell death pathways. Rather than representing a simple consequence of thermal injury, the cellular response to HS reflects an active decision‐making process that determines whether homeostasis can be restored or whether damaged cells must be eliminated.

A central component of this network is the regulation of reactive oxygen species (ROS). Although excessive ROS production is traditionally viewed as a major driver of oxidative damage, accumulating evidence demonstrates that ROS also function as critical signalling molecules that coordinate cellular responses to environmental stress (Chandel, 2010; Finkel, 2011). Consequently, the effects of HS should not be interpreted as a linear process of damage accumulation. Instead, they are better understood as the outcome of a redox‐regulated decision system in which ROS act as central signalling intermediates that determine the balance between adaptation and cell death. Under moderate HS, controlled ROS production activates cytoprotective responses, including antioxidant defences, mitochondrial quality control, and autophagy, thereby promoting cellular resilience and restoration of homeostasis (Paul *et al*., 2008; Chang *et al*., 2022). In contrast, excessive or sustained ROS accumulation overwhelms these protective mechanisms, leading to lipid peroxidation, mitochondrial dysfunction, genomic instability, and activation of regulated cell death (RCD) pathways such as apoptosis, ferroptosis, and pyroptosis (Hasani *et al*., 2022; Qin *et al*., 2021; Su *et al*., 2022; Lei *et al*., 2025).

Importantly, these responses do not operate as isolated pathways but as components of an interconnected regulatory network. Within this framework, autophagy emerges as a pivotal checkpoint that integrates metabolic and stress‐related signals. By controlling the removal of damaged proteins, oxidized lipids, and dysfunctional organelles, autophagy determines whether oxidative stress remains within a manageable range or progresses toward irreversible cellular dysfunction. Therefore, autophagic activity functions as a molecular rheostat that governs the transition between adaptive and deleterious outcomes. When properly regulated, autophagy promotes survival by restoring proteostasis and limiting ROS accumulation. However, when autophagic flux becomes impaired or overwhelmed, oxidative damage intensifies, facilitating the activation of multiple RCD pathways and amplifying cellular injury (Galluzzi *et al*., 2017; Klionsky *et al*., 2021).

Importantly, activation of this redox‐dependent decision network is not uniform across the seminiferous epithelium. Distinct cell populations exhibit substantial differences in metabolic activity, antioxidant capacity, mitochondrial dynamics, and susceptibility to thermal injury. As a result, HS induces highly heterogeneous responses throughout spermatogenesis. Spermatogonia display relative resilience, supported by efficient redox buffering systems and robust stress‐adaptation mechanisms. In contrast, spermatocytes represent a critical vulnerability point due to their elevated metabolic demands, increased mitochondrial ROS generation, and dependence on tightly coordinated meiotic processes, making them particularly prone to apoptosis following thermal insult (Rockett *et al*., 2001; Hikim *et al*., 2003). Post‐meiotic germ cells are especially susceptible to disturbances in membrane integrity, organelle remodelling, and cytoskeletal reorganization, whereas Sertoli cells function as key regulators of the seminiferous microenvironment and may amplify HS‐induced damage through alterations in metabolic support, redox regulation, and inflammatory signalling (Cai *et al*., 2011; Robinson *et al*., 2023).

In this review, we advance the concept that HS regulates spermatogenic fate through a redox‐dependent decision network that integrates ROS signalling, autophagy, and regulated cell death pathways into a unified mechanistic framework (Fig. 1). Rather than considering apoptosis, ferroptosis, pyroptosis, and autophagy as independent responses to thermal stress, we propose that these processes constitute interconnected components of a coordinated cellular decision‐making system. We further identify key regulatory nodes, including ROS thresholds, autophagic flux, mitochondrial dysfunction, lipid peroxidation, and cell type‐specific vulnerabilities, that determine whether germ cells maintain homeostasis or commit to cell death. By reframing HS as a regulator of a redox‐dependent cell fate network rather than a simple inducer of cellular damage, this review provides a mechanistic basis for understanding stage‐specific susceptibility to thermal stress and highlights novel conceptual and therapeutic opportunities for preserving male fertility in an increasingly warming world.

**Fig. 1:**
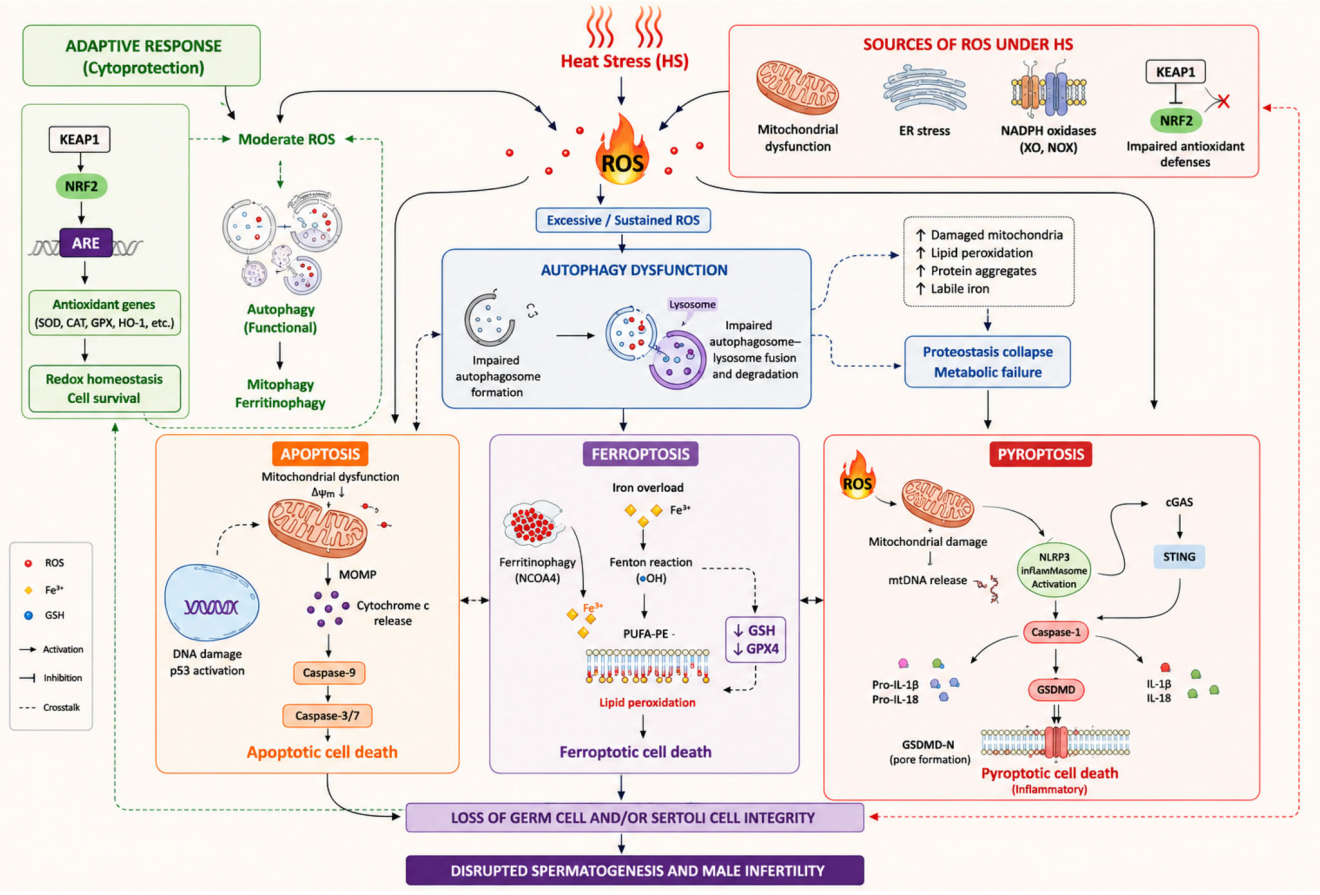
The redox‐dependent cell fate decision network linking heat stress, autophagy, and regulated cell death during spermatogenesis. Heat stress (HS) increases reactive oxygen species (ROS) production through multiple sources, including mitochondrial dysfunction, endoplasmic reticulum (ER) stress, activation of NADPH oxidases, and impairment of antioxidant defences. At moderate levels, ROS activate adaptive responses involving nuclear factor erythroid 2–related factor 2 (NRF2)‐mediated antioxidant signalling and functional autophagy, promoting redox homeostasis, proteostasis, mitochondrial quality control, and cellular survival. However, excessive or sustained ROS accumulation disrupts autophagic flux and antioxidant capacity, leading to proteostasis collapse, mitochondrial dysfunction, iron dysregulation, and lipid peroxidation. This transition represents a critical threshold within the redox‐dependent cell fate decision network, beyond which cells engage regulated cell death (RCD) pathways. Apoptosis is triggered by mitochondrial outer membrane permeabilization (MOMP), cytochrome c release, and caspase activation. Ferroptosis is driven by iron‐dependent lipid peroxidation, enhanced ferritinophagy, glutathione depletion, and glutathione peroxidase 4 (GPX4) inhibition. Pyroptosis is activated through ROS‐induced mitochondrial damage, leading to the release of mitochondrial DNA (mtDNA), activation of the NOD‐, LRR‐ and pyrin domain‐containing protein 3 (NLRP3) inflammasome, caspase‐1 activation, and gasdermin D (GSDMD)‐mediated pore formation. Extensive crosstalk exists among these pathways, with autophagy acting as a central regulatory checkpoint and ROS functioning as the primary quantitative regulator of cell fate. Collectively, the balance between adaptive responses and RCD determines the integrity of germ cells and Sertoli cells, ultimately influencing spermatogenic efficiency and male fertility under thermal stress. Arrow notation: Solid arrows indicate activation or progression of signalling events; T‐shaped lines indicate inhibition; dashed arrows represent indirect interactions, feedback mechanisms, or functional crosstalk; bidirectional arrows denote reciprocal regulation between pathways. Colour coding is used to distinguish biological contexts: green dashed arrows indicate adaptive cytoprotective signalling associated with moderate ROS levels, whereas red dashed arrows indicate deleterious positive‐feedback loops that amplify oxidative stress and cellular injury. Abbreviations: ARE = Antioxidant Response Element; CAT = Catalase; cGAS = Cyclic GMP–AMP Synthase; Δψm = Mitochondrial Membrane Potential; ER = Endoplasmic Reticulum; Fe^3+^ = Ferric iron; GPX4 = Glutathione Peroxidase 4; GSH = Reduced Glutathione; GSDMD = Gasdermin D; HS = Heat Stress; HO‐1 = Heme Oxygenase‐1; KEAP1 = Kelch‐like ECH‐associated Protein 1; MOMP = Mitochondrial Outer Membrane Permeabilization; mtDNA = Mitochondrial DNA; NADPH = Nicotinamide Adenine Dinucleotide Phosphate; NCOA4 = Nuclear Receptor Coactivator 4; NLRP3 = NOD‐, LRR‐ and pyrin domain‐containing Protein 3; NRF2 = Nuclear Factor Erythroid 2–Related Factor 2; Pro‐IL‐1β/18 = Pro‐interleukin‐1β/18; PUFA‐PE = Polyunsaturated Fatty Acid‐containing Phosphatidylethanolamine; RCD = Regulated Cell Death; ROS = Reactive Oxygen Species; SOD = Superoxide Dismutase; STING = Stimulator of Interferon Genes.

## II. ROS AS A QUANTITATIVE REGULATOR OF CELL FATE UNDER HEAT STRESS

ROS occupy a central position within the redox‐dependent cell fate decision network proposed in this review. Rather than acting solely as toxic byproducts of cellular metabolism, ROS function as quantitative signalling molecules that determine whether cells activate adaptive responses or commit to RCD (Chandel, 2010; Finkel, 2011). In this framework, cellular outcomes under HS are governed not simply by the presence of ROS, but by their concentration, duration of exposure, subcellular localization, and the capacity of antioxidant systems to maintain redox homeostasis.

Under physiological conditions, ROS are generated at low levels, primarily through mitochondrial oxidative phosphorylation, where they participate in signalling pathways involved in cellular adaptation, proliferation, and stress responses (Finkel, 2011). This physiological redox state is maintained by a highly coordinated antioxidant network that includes superoxide dismutase (SOD), catalase (CAT), glutathione peroxidases (GPXs), peroxiredoxins, and thioredoxin systems. The expression of many antioxidant defence mechanisms is regulated by the Kelch‐like ECH‐associated protein 1 (KEAP1)–nuclear factor erythroid 2‐related factor 2 (NRF2)–antioxidant response element (ARE) signalling pathway, a central cytoprotective network that functions as a master regulator of cellular redox homeostasis by coordinating the transcription of genes involved in antioxidant defence, detoxification, and cellular stress adaptation (Alnajem & Al‐Maghrebi, 2023).

Heat stress disrupts this equilibrium by simultaneously increasing ROS production and impairing antioxidant defences. Multiple interconnected sources contribute to ROS accumulation within the seminiferous epithelium, including mitochondrial dysfunction, endoplasmic reticulum (ER) stress, activation of NADPH oxidases, and xanthine oxidase activity. Mitochondria appear to represent the primary source of ROS in heat‐stressed germ cells, as thermal injury compromises electron transport chain efficiency and increases electron leakage. Concurrently, ER stress amplifies oxidative damage through activation of the unfolded protein response (UPR), while cytosolic oxidases further exacerbate ROS production (Aitken & Baker, 2006; Turner & Lysiak, 2008; Zhang *et al*., 2012; Karna *et al*., 2020; Hosseini *et al*., 2020; Almarzouq & Al‐Maghrebi, 2023). Suppression of NRF2‐dependent antioxidant signalling further compromises redox buffering capacity, thereby amplifying oxidative stress and reducing cellular resilience (He *et al*., 2022).

Importantly, the biological consequences of ROS accumulation are not binary but occur along a continuum. Moderate increases in ROS activate adaptive pathways that promote cellular survival. These include NRF2‐mediated antioxidant responses, mitochondrial quality‐control mechanisms, and autophagy, which collectively restore proteostasis, remove damaged organelles, and maintain cellular function (Scherz‐Shouval *et al*., 2007; Li *et al*., 2015). Within this adaptive range, ROS function as signalling intermediates that stimulate protective responses and enhance stress tolerance.

However, when ROS production exceeds the buffering capacity of antioxidant and autophagic systems, cells cross a critical redox threshold that fundamentally alters their fate. Excessive or sustained ROS accumulation promotes lipid peroxidation, mitochondrial dysfunction, DNA damage, protein oxidation, and bioenergetic collapse (Aitken & Roman, 2008; Turner & Lysiak, 2008). Simultaneously, oxidative modifications of key autophagy‐related proteins, including Autophagy Related 4 Cysteine Peptidase (ATG4), impair autophagic flux and compromise the removal of damaged cellular components (Scherz‐Shouval *et al*., 2007; Zhang *et al*., 2020). This transition represents a critical tipping point within the redox‐dependent decision network, where adaptive mechanisms become insufficient to restore homeostasis and cellular responses progressively shift toward RCD.

In this context, ROS act as master regulators that coordinate the activation of multiple forms of RCD. Elevated ROS promote mitochondrial apoptosis through loss of mitochondrial membrane potential, cytochrome c release, and caspase activation (Miura *et al*., 2002). In parallel, ROS drive ferroptosis by stimulating iron‐dependent lipid peroxidation and suppressing GPX4 activity, thereby linking oxidative stress to catastrophic membrane damage and metabolic failure (Yang *et al*., 2024; Lei *et al*., 2025). Emerging evidence further indicates that ROS‐induced mitochondrial injury may trigger inflammasome activation and pyroptotic cell death through the release of oxidized mitochondrial DNA, which functions as a damage‐associated molecular pattern (DAMP) capable of activating the NLRP3(NOD‐, LRR‐ and pyrin domain‐containing protein 3) inflammasome and downstream caspase‐1 signalling (Shimada *et al*., 2012; Yu *et al*., 2021). Rather than representing independent pathways, apoptosis, ferroptosis, and pyroptosis can therefore be viewed as interconnected outcomes emerging from a common redox imbalance.

The impact of this redox‐dependent regulation is further shaped by cell type‐specific vulnerabilities within the seminiferous epithelium. Spermatocytes appear particularly susceptible to heat‐induced oxidative stress due to their elevated metabolic demands, intense mitochondrial activity, and dependence on tightly regulated meiotic processes. Consequently, apoptosis is preferentially observed in meiotic germ cells following thermal insult (Rockett *et al*., 2001; Hikim *et al*., 2003). In contrast, spermatogonia exhibit greater resilience, likely reflecting enhanced stress‐adaptation mechanisms and lower metabolic activity. Sertoli cells occupy a unique position within this network, functioning both as regulators of the testicular redox microenvironment and as potential amplifiers of oxidative damage through alterations in metabolic support, antioxidant capacity, and inflammatory signalling. Together, these cell type‐specific differences establish a gradient of susceptibility throughout spermatogenesis, whereby ROS‐dependent signalling determines whether cells adapt, survive, or undergo regulated cell death under heat stress.

Collectively, these observations support the concept that ROS function not merely as mediators of oxidative injury but as quantitative regulators of cell fate. By defining critical redox thresholds that govern the balance between adaptation and cell death, ROS emerge as the central signalling axis of the heat stress response and a key determinant of spermatogenic outcomes.

## III. AUTOPHAGY AS A CENTRAL CHECKPOINT IN REDOX‐DEPENDENT CELL FATE DECISIONS

Within the redox‐dependent cell fate decision network proposed in this review, autophagy occupies a unique regulatory position. Whereas ROS function as quantitative signalling molecules that convey information regarding cellular stress intensity, autophagy acts as a central checkpoint that determines whether cells successfully restore homeostasis or progress toward RCD. Thus, autophagy serves as the principal mechanism through which redox signals are translated into cellular fate decisions, positioning it as a molecular rheostat that governs the transition between adaptation and degeneration (Galluzzi *et al*., 2017; Klionsky *et al*., 2021).

Under moderate HS, ROS activate autophagy through multiple redox‐sensitive signalling pathways. Simultaneously, ROS stimulate NRF2 activation through two complementary mechanisms: direct oxidation of KEAP1 cysteine residues, which impairs NRF2 degradation, and autophagy‐dependent degradation of KEAP1, thereby amplifying antioxidant responses and reinforcing cellular redox homeostasis (Scherz‐Shouval *et al*., 2007; Li *et al*., 2015; Zhu *et al*., 2022). These interconnected processes establish a coordinated adaptive response in which autophagy and antioxidant defences operate synergistically to preserve cellular integrity during thermal stress.

At the molecular level, autophagy is tightly controlled by the intracellular thiol redox network. Reversible oxidation of cysteine residues functions as a regulatory switch that modulates the activity of key autophagy‐related proteins and signalling complexes. Through this mechanism, moderate ROS levels promote autophagosome formation, maturation, and cargo degradation, thereby enhancing proteostasis and facilitating the selective removal of damaged proteins, oxidized lipids, protein aggregates, and dysfunctional mitochondria. This process is particularly important during HS, when mitochondrial injury represents a major source of ROS generation. By eliminating damaged mitochondria through mitophagy, autophagy limits further oxidative stress and prevents the propagation of cellular damage (Filomeni *et al*., 2010; Song, Zhou & Zhou, 2020).

Importantly, the relationship between ROS and autophagy is highly dynamic and bidirectional. Moderate ROS levels stimulate autophagy, whereas effective autophagic flux reduces ROS accumulation by removing damaged cellular components. Together, these mechanisms establish a negative feedback loop that promotes cellular adaptation and enhances stress resilience. As long as this regulatory circuit remains functional, cells retain the capacity to restore homeostasis despite ongoing thermal stress.

However, sustained or excessive ROS accumulation fundamentally alters this balance. Beyond a critical redox threshold, oxidative stress progressively disrupts the autophagic machinery itself. Irreversible oxidation of redox‐sensitive cysteine residues impairs the activity of essential autophagy‐related proteins, including ATG4 and components of the ubiquitin‐like conjugation systems required for light chain 3 (LC3) processing and lipidation (Filomeni *et al*., 2010; Zhu *et al*., 2022). Concurrently, oxidative damage compromises lysosomal integrity through membrane destabilization, impaired acidification, and reduced degradative capacity, thereby disrupting autophagosome–lysosome fusion and blocking autophagic flux (Qi *et al*., 2024). Consequently, the cellular machinery responsible for maintaining proteostasis becomes progressively dysfunctional.

This transition represents a critical tipping point within the redox‐dependent decision network. Once autophagic flux becomes impaired, damaged mitochondria, oxidized proteins, and lipid peroxidation products accumulate, further increasing ROS production and establishing a self‐amplifying cycle of oxidative and proteotoxic stress. Under these conditions, autophagy can no longer function as a protective mechanism, and cells progressively lose their ability to recover from thermal injury. The shift from efficient autophagic flux to autophagic failure therefore constitutes one of the most important decision nodes linking redox imbalance to cell fate determination.

Autophagy also directly influences the activation threshold of multiple RCD pathways. Defective autophagy promotes apoptosis by facilitating mitochondrial dysfunction, cytochrome c release, and caspase activation. Simultaneously, disruption of iron homeostasis and accumulation of oxidized membrane lipids increase susceptibility to ferroptosis, whereas persistent mitochondrial damage and ROS accumulation can potentiate inflammasome activation and pyroptotic signalling (Galluzzi *et al*., 2017; Liu, Wu & Wu, 2023; Qi *et al*., 2024). Conversely, selective forms of autophagy, including mitophagy, ferritinophagy, and lysophagy, actively modulate sensitivity to these death pathways, demonstrating that autophagy is not merely a parallel response but a central regulator of RCD susceptibility (Xu *et al*., 2024).

Within the seminiferous epithelium, the consequences of autophagic dysfunction are further shaped by cell type‐specific requirements. Spermatocytes rely heavily on autophagy‐mediated mitochondrial quality control to sustain meiotic progression and therefore appear particularly vulnerable to disruptions in autophagic flux. Sertoli cells utilize autophagy to recycle cellular components, maintain metabolic support, regulate redox homeostasis, and preserve the seminiferous microenvironment. Consequently, defects in Sertoli cell autophagy may indirectly amplify germ cell damage and compromise spermatogenic function under HS conditions (Shang *et al*., 2016; Pickles *et al*., 2018; Wang *et al*., 2023). Collectively, current evidence supports the concept that autophagy functions as the central checkpoint of the redox‐dependent cell fate decision network. Rather than serving solely as a stress‐response pathway, autophagy determines whether ROS‐induced damage remains compatible with cellular adaptation or progresses toward irreversible dysfunction and regulated cell death. Thus, the integrity of autophagic flux emerges as a critical determinant of spermatogenic resilience under heat stress and a major regulatory node connecting oxidative signalling to cell fate outcomes.

## IV. RCD AS AN OUTCOME OF REDOX IMBALANCE

RCD represents the terminal outcome of the redox‐dependent cell fate decision network activated under HS. Within the conceptual framework proposed in this review, apoptosis, ferroptosis, and pyroptosis should not be viewed as independent responses to thermal injury, but rather as interconnected execution programs that are engaged when adaptive mechanisms fail to restore cellular homeostasis. Thus, RCD pathways constitute the final effector arm of the network, translating persistent redox imbalance, autophagic dysfunction, and metabolic collapse into irreversible cellular outcomes (Galluzzi *et al*., 2017).

The transition from adaptation to cell death occurs when ROS accumulation exceeds the buffering capacity of antioxidant and autophagic systems. As discussed in previous sections, moderate ROS levels activate cytoprotective responses that promote cellular recovery. However, sustained oxidative stress progressively impairs mitochondrial function, disrupts proteostasis, compromises membrane integrity, and destabilizes cellular metabolism. Once these adaptive mechanisms become insufficient, cells cross a critical threshold beyond which recovery is no longer possible and RCD pathways are activated.

Among these pathways, apoptosis represents the predominant response to mitochondrial dysfunction and oxidative DNA damage. Elevated ROS promote mitochondrial outer membrane permeabilization, loss of mitochondrial membrane potential, and cytochrome c release, culminating in activation of the intrinsic caspase cascade (Miura *et al*., 2002). In germ cells, apoptotic susceptibility is closely associated with DNA damage signalling and activation of p53‐dependent pathways, particularly during meiosis. Spermatocytes appear especially vulnerable because of their high metabolic activity, intense mitochondrial function, and strict requirement for genomic integrity during chromosomal recombination. Importantly, impaired autophagic flux further amplifies apoptotic signalling by limiting mitochondrial quality control, promoting ROS accumulation, and sustaining unresolved mitochondrial and endoplasmic reticulum stress (Chitale, Verma & Mugale, 2026). Consequently, apoptosis can be viewed as a primary mechanism through which the seminiferous epithelium eliminates irreversibly damaged germ cells.

Ferroptosis represents a distinct but closely related outcome of redox imbalance. Unlike apoptosis, ferroptosis is driven by iron‐dependent lipid peroxidation and catastrophic membrane damage. Under HS, excessive ROS promote oxidation of polyunsaturated fatty acids within cellular membranes, while depletion of glutathione and inhibition of GPX4 impair the detoxification of lipid peroxides (Li *et al*., 2022; Sun *et al*., 2023). The resulting accumulation of lipid oxidation products drives membrane instability, metabolic dysfunction, and cell death. Autophagy plays a particularly complex role in this process. While basal autophagy limits oxidative damage and promotes cellular survival, selective autophagic pathways such as ferritinophagy increase intracellular iron availability, thereby enhancing susceptibility to ferroptosis (Xu *et al*., 2024). Ferroptosis therefore emerges at the intersection of oxidative stress, lipid metabolism, iron homeostasis, and autophagic regulation, highlighting the extensive crosstalk between adaptive and death‐promoting pathways.

Pyroptotic signalling may also contribute to cellular injury under conditions of severe oxidative stress. Although traditionally associated with innate immune responses, increasing evidence indicates that mitochondrial dysfunction and ROS accumulation can activate inflammatory cell death pathways. Heat‐induced mitochondrial injury promotes the release of oxidized mitochondrial DNA into the cytosol, where it functions as a DAMP capable of activating the NLRP3 inflammasome and downstream caspase‐1 signalling (Shimada *et al*., 2012; Deng *et al*., 2024). Subsequent cleavage of gasdermin D results in membrane pore formation, cell swelling, and release of pro‐inflammatory mediators. Within the testis, this pathway may be particularly relevant in Sertoli cells, where inflammasome activation has the potential to disrupt blood–testis barrier integrity and amplify local inflammatory responses, thereby extending damage beyond individual cells.

Importantly, these RCD pathways exhibit extensive mechanistic overlap and should be considered components of an integrated response rather than isolated biological processes. Mitochondrial dysfunction can simultaneously initiate apoptotic and inflammatory signalling, while ROS‐induced lipid peroxidation serves as a common driver of ferroptotic susceptibility. Likewise, autophagic failure acts as a major convergence point that amplifies oxidative stress, compromises organelle quality control, and lowers the activation threshold for multiple death pathways. Consequently, the specific form of RCD observed under HS is not determined by a single molecular trigger but emerges from the cumulative interaction among ROS dynamics, metabolic status, organelle integrity, and cellular adaptive capacity.

The engagement of individual RCD pathways is further influenced by cell type‐specific characteristics within the seminiferous epithelium. Spermatocytes predominantly undergo apoptosis because of their sensitivity to oxidative DNA damage and mitochondrial dysfunction. Ferroptotic susceptibility may be enhanced in cells characterized by elevated polyunsaturated lipid content and increased iron availability. Sertoli cells occupy a central regulatory position, as they can both modulate and propagate stress signals through alterations in redox homeostasis, inflammatory signalling, and metabolic support. These differences establish a functional hierarchy of vulnerability in which distinct cell populations engage different death programs despite sharing common upstream stress signals.

Collectively, current evidence supports the concept that apoptosis, ferroptosis, and pyroptosis represent interconnected execution programs downstream of a common redox‐dependent decision network. Their activation reflects the progressive failure of adaptive mechanisms to contain oxidative stress and maintain cellular homeostasis. Understanding how these pathways converge and interact provides a mechanistic framework for identifying critical intervention points aimed at preserving spermatogenesis and male fertility under conditions of thermal stress.

## V. REDOX‐DEPENDENT REGULATION OF SPERMATOGENESIS UNDER HEAT STRESS

Building on the redox‐dependent cell fate decision network proposed in this review, the impact of HS on spermatogenesis can be understood as a stage‐specific manifestation of a conserved regulatory axis integrating ROS dynamics, mitochondrial function, autophagic flux, and RCD pathways. Within this framework, the biological consequences of HS are not uniformly distributed throughout spermatogenesis but emerge from intrinsic differences in metabolic activity, mitochondrial dependency, antioxidant capacity, and proteostatic demands among distinct germ cell populations. These characteristics establish cell type‐specific thresholds of stress tolerance that determine whether cells successfully adapt to thermal challenge or progress toward RCD.

To better understand these differential responses, spermatogenesis can be functionally divided into three major developmental phases: the mitotic phase (spermatogonia), the meiotic phase (spermatocytes), and post‐meiotic differentiation (spermiogenesis and Spermiation; Fig. 2). Each stage is characterized by unique energetic requirements and redox constraints, resulting in distinct sensitivities to oxidative stress. Consequently, the response to HS reflects not only the magnitude of ROS accumulation but also the capacity of individual cell types to activate antioxidant defences, maintain mitochondrial quality control, and preserve efficient autophagic flux.

**Fig. 2:**
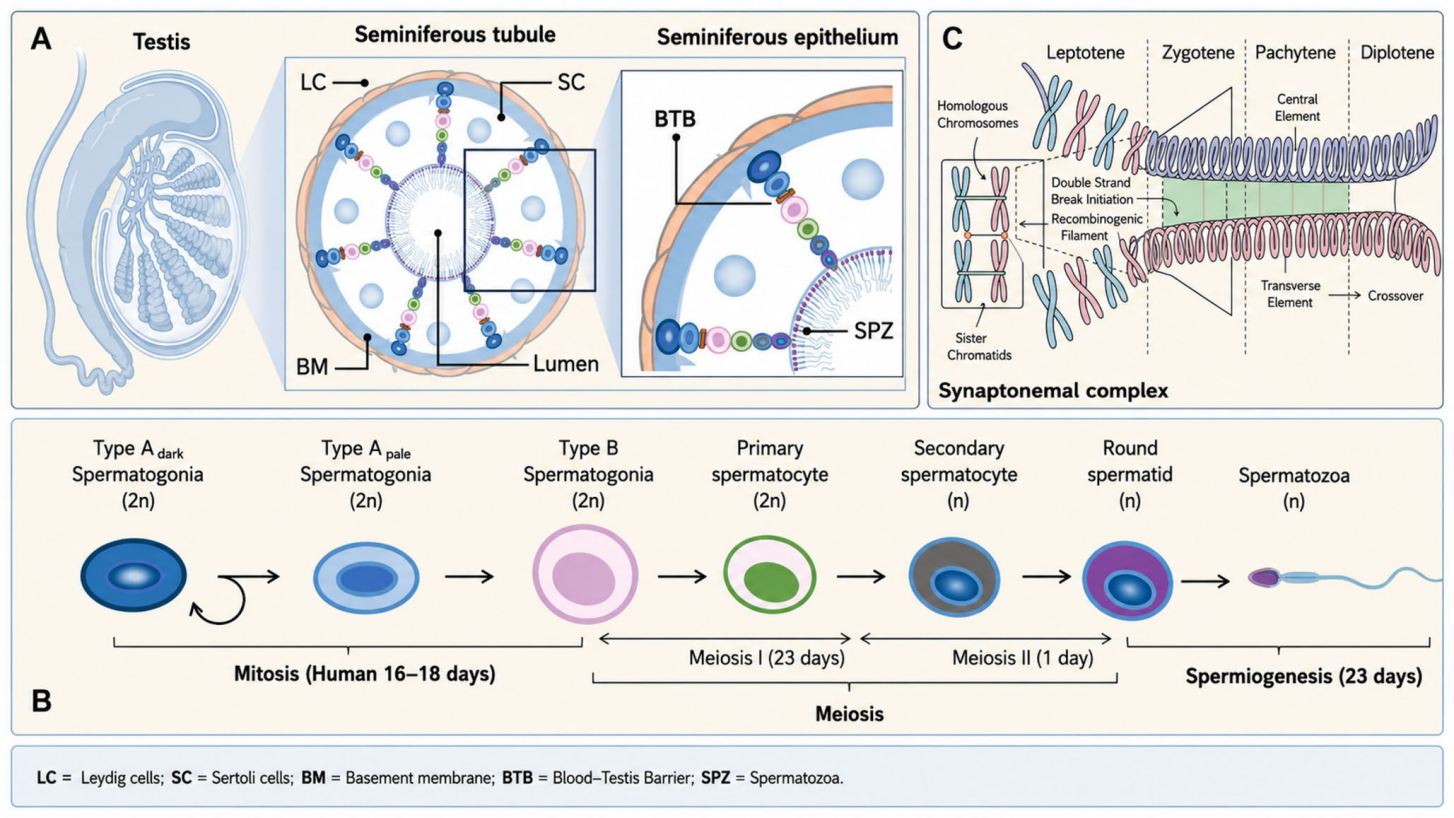
Testicular architecture and cellular dynamics of human spermatogenesis. (A) Schematic representation of testicular organization, highlighting the seminiferous tubules, the site of spermatogenesis. The cross‐sectional view of a seminiferous tubule illustrates the radial arrangement of germ cells at different developmental stages, supported by Sertoli cells (SC). Leydig cells (LC), located within the interstitial compartment, are responsible for testosterone production. The basement membrane (BM) delineates the seminiferous epithelium and provides the niche for spermatogonial stem cells. The enlarged inset depicts the blood–testis barrier (BTB), formed by specialized junctional complexes between adjacent Sertoli cells, which separates the basal and adluminal compartments and protects meiotic and post‐meiotic germ cells from immune surveillance. Mature spermatozoa (SPZ) are released into the lumen upon completion of spermatogenesis. (B) Stages of spermatogenic development. The mitotic phase begins with Type A_dark_ spermatogonia (Ad), a quiescent reserve stem cell population, which give rise to Type A_pale_ spermatogonia (Ap) and subsequently Type B spermatogonia, all of which remain diploid (2n). These cells differentiate into primary spermatocytes (2n) and enter meiosis. Following meiosis I (approximately 23 days), primary spermatocytes generate haploid (n) secondary spermatocytes, which rapidly complete meiosis II (approximately 1 day) to produce round spermatids (n). Spermatids subsequently undergo spermiogenesis (approximately 23 days), a highly specialized differentiation process involving extensive morphological and biochemical remodelling without further cell division, ultimately giving rise to mature spermatozoa. (C) Structural organization of the synaptonemal complex during prophase I of meiosis. During leptotene, chromosomes begin to condense and programmed DNA double‐strand breaks are initiated. During zygotene, homologous chromosomes align and synapsis begins through the assembly of the transverse filaments and central elements of the synaptonemal complex. Pachytene is characterized by complete synapsis and active homologous recombination, including crossing‐over events. During diplotene, the synaptonemal complex progressively disassembles, allowing homologous chromosomes to separate while remaining connected at chiasmata. These coordinated meiotic events ensure accurate chromosome segregation, promote genetic diversity, and preserve germline genome integrity.

In this context, the balance between ROS production and autophagy‐dependent proteostasis acts as a central rheostat regulating germ cell fate. When redox homeostasis is maintained, adaptive responses support cellular survival and developmental progression. Conversely, when oxidative stress exceeds the buffering capacity of antioxidant and autophagic systems, cells cross critical thresholds that trigger mitochondrial dysfunction, proteostatic collapse, and activation of RCD pathways. Thus, stage‐specific susceptibility to HS emerges as a direct consequence of how individual germ cell populations engage the redox‐dependent decision network, ultimately determining spermatogenic efficiency and reproductive competence under thermal stress (Fig. 3).

**Fig. 3:**
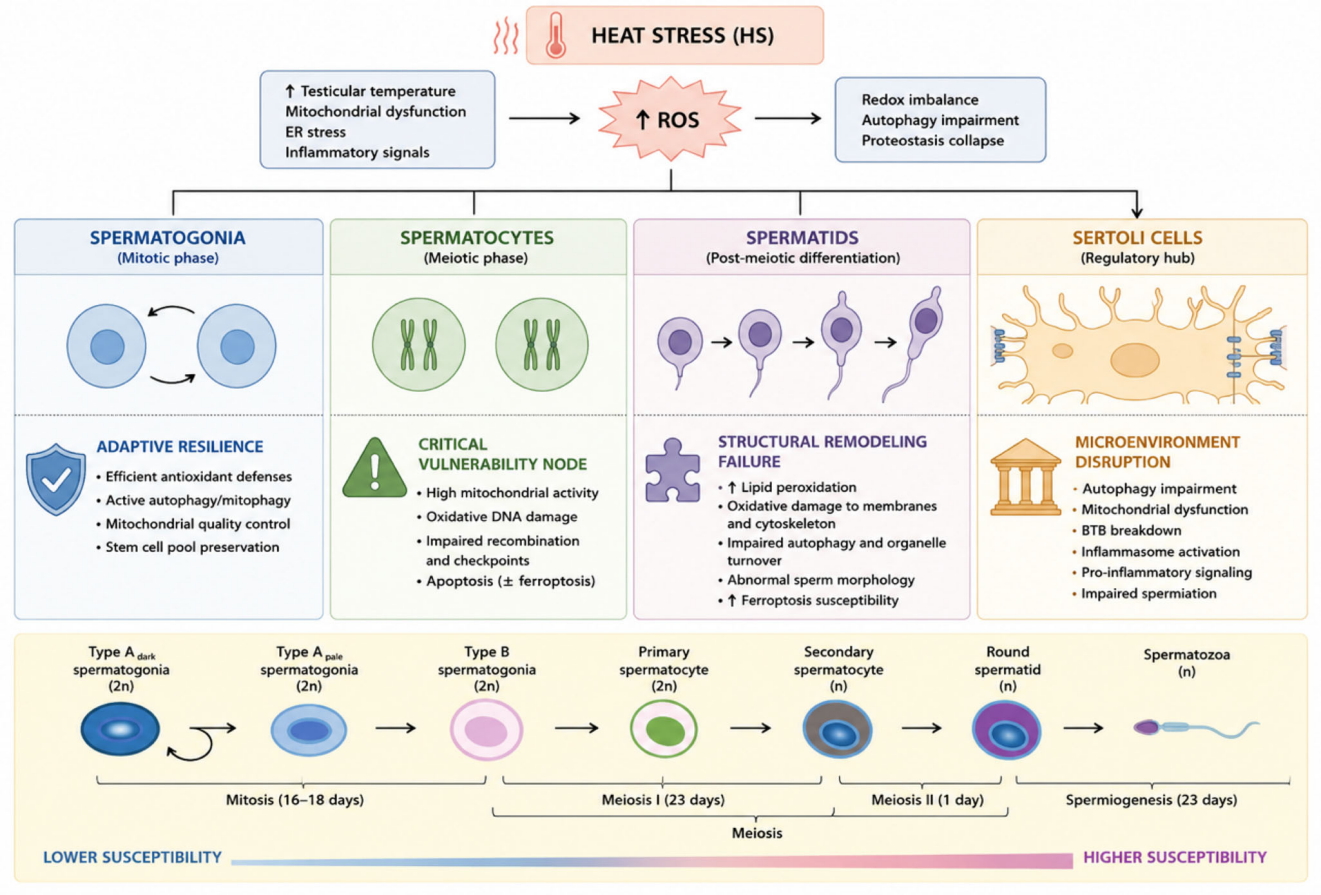
Stage‐specific responses of germ cells and Sertoli cells to heat stress within the redox‐dependent cell fate decision network. Heat stress (HS) increases reactive oxygen species (ROS) production through mitochondrial dysfunction, endoplasmic reticulum (ER) stress, activation of oxidant‐generating pathways, and impairment of antioxidant defences. The resulting redox imbalance differentially affects cell populations within the seminiferous epithelium according to their metabolic demands, mitochondrial dependency, and capacity to maintain autophagy‐dependent proteostasis. Spermatogonia represent the adaptive end of the redox continuum and exhibit relative resilience due to efficient antioxidant defences, active mitophagy, and preservation of the stem cell pool. Spermatocytes constitute a critical vulnerability node characterized by high mitochondrial activity, increased ROS production, oxidative DNA damage, impaired meiotic recombination, and predominant activation of apoptotic pathways. During post‐meiotic differentiation, spermatids are particularly susceptible to lipid peroxidation and disruption of autophagy‐mediated organelle remodelling, resulting in defective spermiogenesis and increased ferroptotic susceptibility. Sertoli cells function as the principal regulatory hub of the seminiferous microenvironment, coordinating redox homeostasis, metabolic support, blood–testis barrier integrity, and autophagic activity. Under severe or sustained oxidative stress, Sertoli cell dysfunction amplifies oxidative damage through mitochondrial impairment, inflammasome activation, disruption of cell junctions, and loss of microenvironmental control. Together, these stage‐specific responses establish a gradient of susceptibility to HS, in which the balance between ROS accumulation and adaptive mechanisms determines whether spermatogenesis is preserved or progresses toward cellular dysfunction and impaired fertility.

### (1) Mitotic phase: relative resilience and preservation of the stem cell pool

Within the redox‐dependent cell fate decision network, spermatogonia represent the most resilient germ cell population during HS. Their relative resistance reflects a combination of lower metabolic demand, reduced dependence on mitochondrial oxidative phosphorylation, and consequently lower basal mitochondrial ROS production compared with more differentiated germ cells (Rockett *et al*., 2001; Hikim *et al*., 2003; Wang *et al*., 2015). As a result, spermatogonia occupy a region of the redox continuum that favors adaptation rather than activation of RCD, allowing preservation of the spermatogonial stem cell pool even under moderate thermal challenge.

This adaptive capacity is supported by efficient antioxidant defences and robust autophagy‐dependent proteostasis. Moderate increases in ROS are effectively buffered through coordinated activation of redox regulatory pathways, preventing excessive oxidative damage while maintaining physiological redox signalling. Autophagy, particularly mitophagy, plays a pivotal role in this process by preserving mitochondrial quality control and limiting ROS accumulation through the selective removal of damaged mitochondria and oxidized cellular components (Scherz‐Shouval *et al*., 2007; Li *et al*., 2015). Consequently, efficient autophagic flux stabilizes intracellular redox homeostasis and reinforces cellular resilience during thermal stress.

Importantly, the relative resistance of spermatogonia does not imply complete protection from HS‐induced injury. Rather, it reflects a greater capacity to maintain redox homeostasis and proteostatic integrity before reaching the critical threshold that triggers RCD. Under conditions of prolonged or severe HS, persistent ROS accumulation progressively compromises mitochondrial function, overwhelms antioxidant defences, and impairs autophagic flux. As adaptive capacity declines, cells become increasingly susceptible to mitochondrial dysfunction, oxidative DNA damage, and activation of apoptotic pathways. In parallel, sustained lipid peroxidation and disruption of glutathione‐dependent antioxidant systems may increase ferroptotic susceptibility (Zhang *et al*., 2012; Li *et al*., 2022).

Although spermatogonia are considerably less sensitive to thermal injury than meiotic germ cells, chronic oxidative stress can ultimately deplete the spermatogonial stem cell pool, thereby compromising the regenerative capacity of the seminiferous epithelium and impairing long‐term spermatogenic recovery (Lue *et al*., 2002; Rahman *et al*., 2011). Preservation of this population is therefore essential not only for immediate cellular survival but also for restoration of spermatogenesis following thermal insult.

Collectively, the mitotic phase represents the adaptive end of the redox‐dependent decision network, where efficient coordination among ROS regulation, mitochondrial quality control, antioxidant defences, and autophagy‐dependent proteostasis maintains stem cell integrity and promotes resilience to transient thermal stress. The ability of spermatogonia to remain below the threshold for widespread RCD highlights the central importance of redox homeostasis in preserving the foundation of spermatogenic renewal.

### (2) Meiotic phase: a critical vulnerability node within the redox‐dependent decision network

The meiotic phase represents the most vulnerable stage of spermatogenesis to HS, positioning spermatocytes as a critical node within the redox‐dependent cell fate decision network. Unlike spermatogonia, which occupy the adaptive end of the redox continuum, spermatocytes operate close to the threshold separating cellular adaptation from RCD. This heightened susceptibility arises from their elevated metabolic demand, strong dependence on mitochondrial oxidative phosphorylation, and the complex molecular processes required for meiotic progression, including homologous recombination, DNA repair, chromosome synapsis, and chromatin remodelling (Rockett *et al*., 2001; Hikim *et al*., 2003; Wang *et al*., 2015).

These characteristics create a unique biological context in which even modest perturbations in redox homeostasis can have profound consequences. Mitochondrial activity is markedly increased during meiosis, resulting in enhanced ROS generation and a greater dependence on antioxidant defences and mitochondrial quality‐control mechanisms. Consequently, spermatocytes exist in a relatively narrow physiological window between productive redox signalling and oxidative injury. Within the framework proposed here, this places meiotic germ cells near the upper limit of ROS tolerance, making them particularly sensitive to HS‐induced redox imbalance.

Elevated ROS levels disrupt multiple aspects of meiotic progression. Oxidative DNA damage interferes with homologous recombination and compromises the accurate repair of programmed DNA double‐strand breaks, thereby activating DNA damage response pathways and meiotic checkpoints. Simultaneously, mitochondrial dysfunction amplifies ROS production and promotes genomic instability, creating a self‐reinforcing cycle that further impairs chromosome dynamics and meiotic progression (Hikim *et al*., 2003; Wang *et al*., 2017). As these defects accumulate, spermatocytes frequently undergo meiotic arrest or are eliminated through apoptosis, reflecting the stringent quality‐control mechanisms that preserve genomic integrity during gametogenesis.

Autophagy functions as a critical regulatory checkpoint during this stage. Under moderate stress conditions, efficient autophagic flux supports mitochondrial quality control, preserves proteostasis, and facilitates successful meiotic progression. Through selective removal of damaged mitochondria and oxidized cellular components, autophagy limits ROS accumulation and maintains cellular homeostasis despite ongoing thermal challenge. However, when ROS production exceeds the buffering capacity of adaptive systems, oxidative stress progressively impairs autophagic flux, leading to defective organelle turnover and accumulation of dysfunctional mitochondria, protein aggregates, and oxidized macromolecules (Filomeni *et al*., 2010; Zhang *et al*., 2012). This shift from functional autophagy to autophagic failure represents a critical tipping point within the redox‐dependent decision network.

Experimental evidence supports the importance of this interaction, as modulation of autophagy‐related genes such as ATG7 influences the susceptibility of spermatocyte‐derived cells to HS‐induced apoptosis, highlighting the intimate crosstalk between autophagic regulation and cell death pathways under oxidative stress (Zhang *et al*., 2012). Furthermore, disruption of post‐transcriptional regulatory structures, including chromatoid bodies and piRNA‐associated pathways, has been linked to ROS accumulation and may further compromise germ cell survival by impairing genome stability and transcriptomic regulation during meiosis (Da Ros *et al*., 2017; Cappucci *et al*., 2019; Mehta *et al*., 2024).

Once the critical redox threshold is exceeded, adaptive mechanisms rapidly collapse and RCD pathways become engaged. Apoptosis represents the predominant outcome, driven by oxidative DNA damage, mitochondrial dysfunction, and activation of intrinsic cell death signalling. However, increasing evidence suggests that ferroptotic mechanisms may also contribute to spermatocyte loss under conditions of severe oxidative stress, particularly when lipid peroxidation and antioxidant failure become prominent features of the cellular response. Thus, the meiotic phase constitutes a decisive vulnerability point within the redox‐dependent decision network, where the convergence of elevated ROS production, stringent genomic surveillance, mitochondrial dependency, and autophagic regulation creates a uniquely fragile cellular state. This vulnerability largely explains the preferential and rapid loss of spermatocytes observed following thermal insult and identifies meiosis as a major determinant of heat stress‐induced impairment of spermatogenesis.

### (3) Post‐meiotic differentiation: structural vulnerability and redox‐dependent failure of spermatid maturation

Post‐meiotic differentiation represents a distinct stage within the redox‐dependent cell fate decision network, characterized by extensive cellular remodelling rather than active proliferation or meiotic progression. During spermiogenesis, haploid spermatids undergo profound morphological and biochemical transformations, including chromatin condensation, acrosome biogenesis, flagellum assembly, mitochondrial reorganization, and elimination of excess cytoplasm. These processes require precise coordination of membrane trafficking, organelle turnover, protein quality control, and cytoskeletal remodelling, rendering post‐meiotic germ cells particularly vulnerable to disturbances in redox homeostasis (Shang *et al*., 2016; Yang *et al*., 2017; Finetti *et al*., 2020).

Unlike spermatocytes, which frequently respond to HS through rapid activation of RCD, post‐meiotic germ cells often exhibit a different outcome. Rather than undergoing immediate elimination, spermatids tend to accumulate structural and functional defects that compromise their maturation and subsequent fertilizing capacity. Within the framework proposed here, this stage occupies a region of the redox continuum in which oxidative stress primarily disrupts differentiation programs and cellular architecture rather than directly triggering widespread cell death.

Elevated ROS levels interfere with multiple aspects of spermatid maturation. Oxidative damage to proteins, membranes, and cytoskeletal components disrupts the highly coordinated remodelling events required for successful spermiogenesis. In particular, lipid peroxidation of polyunsaturated phospholipids compromises membrane fluidity and integrity, adversely affecting acrosome formation, flagellar assembly, and organelle organization (Aitken, 2017; Ozturk, Steger & Schagdarsurengin, 2017). Mitochondrial remodelling, which is essential for the acquisition of mature sperm architecture and energy metabolism, is also highly sensitive to oxidative stress. Persistent ROS production therefore destabilizes the intracellular environment necessary for coordinated differentiation and contributes to the generation of structurally abnormal spermatozoa.

Autophagy plays a fundamental role in maintaining developmental progression during this stage. Efficient autophagic flux supports cytoplasmic reduction, organelle remodelling, residual body formation, and mitochondrial quality control, all of which are essential for sperm maturation. Through selective degradation of excess cytoplasmic components and damaged organelles, autophagy preserves proteostasis and facilitates the acquisition of the highly specialized architecture characteristic of mature spermatozoa. However, sustained oxidative stress progressively impairs autophagic flux, resulting in defective organelle turnover, incomplete cytoplasmic clearance, and accumulation of damaged cellular structures (Ktistakis & Tooze, 2016; Shang *et al*., 2016). Consequently, disruption of autophagy compromises spermatid remodelling and contributes to the emergence of morphological abnormalities frequently associated with thermal stress.

In contrast to the meiotic phase, where apoptosis predominates, post‐meiotic germ cells may be particularly susceptible to ferroptosis‐related mechanisms. The abundance of polyunsaturated fatty acids within developing sperm membranes, together with the high demand for membrane remodelling, creates an environment that is intrinsically vulnerable to lipid peroxidation. Under HS, disruption of glutathione‐dependent antioxidant systems, including reduced GPX4 activity, combined with increased availability of redox‐active iron, amplifies oxidative membrane damage and enhances ferroptotic susceptibility (Aitken *et al*., 2017; Sun *et al*., 2023). Although complete ferroptotic cell death may not always occur, ferroptosis‐associated molecular events can significantly impair membrane integrity and compromise sperm function.

As a result, the biological consequences of HS during post‐meiotic differentiation differ fundamentally from those observed during meiosis. Whereas spermatocyte loss primarily reflects activation of cell death pathways, spermatid injury is often manifested as defective maturation, abnormal morphology, impaired motility, compromised chromatin packaging, and reduced fertilization capacity. Thus, post‐meiotic differentiation represents the structural vulnerability end of the redox‐dependent decision network, where oxidative stress disrupts the processes required for sperm assembly and functional specialization. Failure to maintain redox homeostasis, membrane integrity, and autophagy‐dependent quality control ultimately results in the production of morphologically and functionally compromised spermatozoa, thereby reducing reproductive fitness under conditions of thermal stress.

### (4) Spermiation and Sertoli cell dysfunction: failure of the regulatory hub controlling the seminiferous microenvironment

Spermiation represents the final stage of spermatogenesis and depends on the precise coordination between elongating spermatids and Sertoli cells. Successful release of mature spermatozoa requires tightly regulated cytoskeletal remodelling, membrane trafficking, adhesion dynamics, and coordinated communication between germ cells and their supporting microenvironment. Within the redox‐dependent cell fate decision network, this stage is uniquely dependent on the capacity of Sertoli cells to maintain redox homeostasis and preserve the structural and functional integrity of the seminiferous epithelium. Consequently, spermiation failure under HS often reflects not only direct germ cell injury but also disruption of the regulatory mechanisms governing the seminiferous microenvironment.

Elevated ROS levels interfere with multiple processes required for spermatid release. Oxidative stress disrupts cytoskeletal organization and impairs the dynamic restructuring of adhesion complexes within the apical ectoplasmic specialization, a specialized junctional structure that anchors elongating spermatids to Sertoli cells. Redox‐dependent alterations in actin dynamics, adhesion proteins, and signalling pathways compromise spermatid disengagement and frequently result in retention of mature spermatids within the seminiferous epithelium (Cheng & Mruk, 2010). As a consequence, sperm output is reduced despite completion of much of the preceding differentiation program.

Sertoli cells occupy a central regulatory position within this process. Beyond their structural support functions, they actively control the redox status, metabolic environment, nutrient availability, and quality‐control systems that sustain germ cell development. Under moderate stress conditions, Sertoli cells contribute to cellular adaptation through antioxidant activity, metabolic cooperation, and autophagy‐dependent recycling pathways that maintain proteostasis and preserve blood–testis barrier (BTB) integrity (Riera *et al*., 2012; Chu, Deng & Fang, 2025). Through these functions, Sertoli cells act as a buffering system that limits ROS propagation and protects developing germ cells from oxidative injury.

However, prolonged or severe HS can overwhelm this protective capacity. Sustained ROS accumulation progressively impairs autophagic flux, compromises mitochondrial function, and disrupts the ability of Sertoli cells to maintain redox homeostasis (Cao *et al*., 2023; Chu *et al*., 2025). As adaptive mechanisms fail, deterioration of BTB integrity and junctional organization increases the vulnerability of germ cells to oxidative damage and disrupts the highly coordinated environment required for spermatogenesis. Within the framework proposed here, this transition represents a critical shift in which Sertoli cells change from buffering oxidative stress to amplifying its effects throughout the seminiferous epithelium.

In addition to structural dysfunction, increasing evidence suggests that oxidative stress may activate inflammatory signalling pathways within Sertoli cells. Mitochondrial injury and release of oxidized mitochondrial DNA can stimulate pattern‐recognition pathways and promote inflammasome activation, leading to caspase‐1 signalling and production of pro‐inflammatory mediators (Deng *et al*., 2024; Chu *et al*., 2025). These responses further compromise Sertoli cell function and establish a pro‐inflammatory microenvironment that exacerbates tissue injury. Consequently, oxidative stress not only disrupts spermiation directly but also propagates damage through inflammatory amplification mechanisms.

Unlike earlier stages of spermatogenesis, where cell‐autonomous responses largely determine cellular outcomes, the consequences of HS during spermiation emerge predominantly from failure of microenvironmental regulation. Thus, Sertoli cells function as the principal regulatory hub of the redox‐dependent decision network, integrating oxidative signalling, metabolic support, autophagic activity, barrier integrity, and inflammatory responses. When this regulatory hub remains functional, spermatogenesis can proceed despite moderate thermal challenge. Conversely, when Sertoli cell homeostatic capacity collapses, oxidative stress propagates throughout the seminiferous epithelium, leading to defective spermiation, reduced sperm output, and compromised fertility. The final outcome of HS therefore depends not only on the susceptibility of individual germ cells but also on the ability of Sertoli cells to coordinate and preserve the seminiferous microenvironment.

## VI. THERAPEUTIC MODULATION OF THE REDOX‐DEPENDENT DECISION NETWORK UNDER HEAT STRESS

Given the central role of ROS in regulating cell fate under HS, modulation of redox balance represents a promising strategy to preserve spermatogenesis and male fertility. Within the framework proposed in this review, antioxidant interventions should not be viewed merely as ROS scavengers, but rather as modulators of a redox‐dependent decision network that integrates mitochondrial function, autophagic flux, inflammatory signalling, and RCD pathways (Finkel, 2011; Sies, Berndt & Jones, 2017). Rather than acting through a single mechanism, effective therapeutic strategies converge on key regulatory nodes that determine whether cells maintain homeostasis or transition toward dysfunction and cell death. Heat stress induces a coordinated disruption of testicular homeostasis characterized by mitochondrial dysfunction, excessive ROS production, lipid peroxidation, activation of inflammatory pathways, autophagic imbalance, and germ cell loss, ultimately impairing spermatogenesis and reducing fertility.

Consistent with this integrative framework, a growing number of antioxidant interventions have demonstrated protective effects in experimental models of testicular HS (Table 1). These compounds encompass diverse chemical classes, including indoleamines (melatonin), osmoprotectants (betaine), trace elements (zinc and selenium), polyphenols (resveratrol), mitochondrial antioxidants (α‐lipoic acid), lipid‐phase antioxidants (sesame seed oil), and bioactive diterpenoids (tanshinone IIA). Despite their chemical diversity, these interventions converge mechanistically on the attenuation of oxidative stress, preservation of mitochondrial function, modulation of inflammatory signalling, maintenance of autophagic balance, and suppression of RCD pathways. Collectively, these observations reinforce the concept that protection against HS is achieved through restoration of network integrity rather than through simple neutralization of ROS.

**Table 1:** Antioxidant interventions experimentally validated under heat stress (HS) in male reproductive models. This table summarizes antioxidant compounds with direct experimental evidence under heat stress conditions, highlighting their primary mechanisms, molecular targets, and protective effects on testicular function and spermatogenesis. The molecular pathways involved include redox regulators such as nuclear factor erythroid 2–related factor 2 (NRF2) and heme oxygenase‐1 (HO‐1); antioxidant enzymes including superoxide dismutase (SOD), catalase (CAT), and glutathione peroxidases (GPX1/GPX4); apoptosis‐related proteins such as BCL2‐associated X protein (BAX), B‐cell lymphoma 2 (BCL2), and caspase‐3; and autophagy markers including microtubule‐associated protein 1 light chain 3 (LC3) and sequestosome 1 (p62). Additional targets include inflammatory mediators [interleukin‐1 beta (IL‐1β) and tumour necrosis factor alpha (TNF‐α)], mitochondrial and oxidative stress markers [reactive oxygen species (ROS) and malondialdehyde (MDA)], steroidogenic enzymes [3β‐hydroxysteroid dehydrogenase (3β‐HSD) and 17β‐hydroxysteroid dehydrogenase (17β‐HSD)], and signalling pathways such as transforming growth factor beta 1 (TGF‐β1)/Sma‐ and Mothers Against Decapentaplegic Homolog (SMAD) signalling. Collectively, these interventions converge on the modulation of oxidative stress, apoptosis, and cellular homeostasis, supporting a redox‐dependent regulation of cell fate under heat stress. ALA = α‐Lipoic Acid; BHMT = Betaine‐Homocysteine S‐Methyltransferase; Ki‐67 = Marker of Cellular Proliferation Ki‐67; PCNA = Proliferating Cell Nuclear Antigen.

Antioxidant	Primary mechanism	Molecular targets	Effect under HS	References
Melatonin	ROS scavenger + redox modulation	NRF2/HO‐1, SOD, CAT, GPX4, BAX/BCL2, LC3, p62	Reduces oxidative stress, inhibits apoptosis and ferroptosis, restores autophagic flux, improves spermatogenesis	Liu *et al*., 2022; Zhang *et al*., 2020; Qin *et al*., 2021; Guo *et al*., 2024; Lei *et al*., 2025
Betaine	Osmoprotection + redox balance	BHMT, SOD, CAT	Reduces oxidative damage and improves spermatogenesis	Shadmehr *et al*., 2018
Zinc	Antioxidant cofactor	Cu/Zn‐SOD	Reduces testicular oxidative stress	Cao *et al*., 2015
Resveratrol	Polyphenolic antioxidant with anti‐inflammatory, antiapoptotic, and pro‐proliferative effects	SOD, CAT, GPX, MDA, nitrite, IL‐1β, TNF‐α, BAX, caspase‐3, Ki‐67, PCNA, 3β‐HSD, 17β‐HSD	Mitigates HS‐induced testicular injury, restores antioxidant defences, reduces inflammation and apoptosis, improves sperm quality, reproductive hormones, and fertility outcomes	Hafez *et al*., 2025
Alpha‐lipoic acid	Mitochondrial antioxidant	ROS, NRF2	Reduces apoptosis and improves fertility	Santos *et al*., 2025; Hashemi *et al*., 2026
Sesame seed oil	Lipid antioxidant	Lipid peroxidation pathways	Improves sperm quality; synergistic with ALA	Hashemi *et al*., 2026
Selenium	Selenoprotein antioxidant	GPX1, GPX4	Improves semen quality and redox status	Kaur & Bansal, 2014; Hosny *et al*., 2020; Fadl *et al*., 2024
Tanshinone IIA	Anti‐fibrotic + antioxidant	TGFβ1/SMAD pathway	Protects testes from HS injury	Bai *et al*., 2023

Among the available interventions, melatonin provides particularly strong mechanistic support for the redox‐dependent model proposed here. Experimental evidence demonstrates that melatonin attenuates lipid peroxidation, enhances antioxidant enzyme activity, and suppresses germ cell apoptosis through modulation of BAX/BCL2 (BCL2‐associated X protein/B‐cell lymphoma 2) balance and caspase activation (Liu *et al*., 2022). More recent studies further reveal that melatonin acts as a multi‐target regulator capable of restoring autophagic flux, reducing intracellular iron accumulation, and upregulating GPX4, thereby limiting ferroptotic signalling and preserving spermatocyte integrity under HS conditions (Lei *et al*., 2025). These findings position melatonin as a key modulator of the autophagy–ferroptosis axis and illustrate how therapeutic interventions can influence multiple components of the redox‐dependent decision network simultaneously.

Similarly, resveratrol exerts broad protective effects that extend beyond classical antioxidant activity. In experimental models of scrotal hyperthermia, HS induces marked reductions in antioxidant defences, including SOD, GPX, and catalase CAT, while increasing lipid peroxidation, nitrosative stress, inflammatory signalling (TNF‐α and IL‐1β), and apoptotic markers such as Bax and caspase‐3. These alterations are accompanied by impaired steroidogenesis, histopathological damage, and reduced sperm quality. Resveratrol treatment significantly attenuates these effects by restoring redox homeostasis, suppressing inflammatory and apoptotic pathways, improving testicular architecture, and enhancing reproductive outcomes (Hafez *et al*., 2025). These findings emphasize that antioxidant interventions frequently operate as system‐level regulators capable of targeting multiple interconnected stress‐response pathways.

Additional antioxidant strategies further support this integrative model. Betaine improves cellular osmotic balance and redox homeostasis, thereby preserving spermatogenesis under thermal stress (Shadmehr *et al*., 2018). Zinc contributes to antioxidant defence through its role as a cofactor of enzymes such as Cu/Zn‐superoxide dismutase and participates in the maintenance of testicular redox balance (Cao *et al*., 2015). Selenium, through its incorporation into selenoproteins including GPX1 and GPX4, plays a central role in controlling lipid peroxidation and ferroptotic signalling, thereby preserving sperm quality under HS conditions (Kaur & Bansal, 2014; Hosny *et al*., 2020). Likewise, α‐lipoic acid improves mitochondrial function, suppresses oxidative and endoplasmic reticulum stress, and reduces ROS accumulation under heat stress (Xiong *et al*., 2020), whereas sesame seed oil provides lipid‐phase antioxidant protection and may act synergistically with α‐lipoic acid to improve fertility outcomes under thermal stress (Hashemi *et al*., 2026). Tanshinone IIA further expands this therapeutic framework by modulating transforming growth factor beta 1 (TGF‐β1)/Sma and Mad‐related protein (SMAD) signalling, linking oxidative stress to fibrotic remodelling and structural alterations within the testis (Bai *et al*., 2023).

Importantly, many of these interventions influence autophagy, reinforcing its role as a central rheostat within the redox‐dependent decision network. As discussed in previous sections, moderate ROS levels stimulate adaptive autophagic responses that support mitochondrial quality control, proteostasis, and cellular resilience. Conversely, excessive ROS disrupt autophagic flux, leading to accumulation of damaged organelles, impaired mitochondrial turnover, and amplification of intracellular stress. Restoration of autophagic balance, as observed in melatonin‐treated models, represents a critical mechanism through which antioxidant strategies prevent the transition from adaptation to RCD under HS conditions (Lei *et al*., 2025). These findings are consistent with established redox‐dependent regulation of autophagy through reversible oxidation of thiol groups in autophagy‐related proteins, which function as molecular switches linking oxidative stress to autophagic commitment (Filomeni *et al*., 2010).

A key implication of the framework proposed in this review is that successful therapeutic interventions should not aim to indiscriminately suppress ROS production, but rather to restore the adaptive capacity of the redox‐dependent decision network. Heat stress‐induced infertility emerges not simply from oxidative damage, but from the progressive failure of interconnected regulatory systems responsible for maintaining redox homeostasis, mitochondrial quality control, autophagic competence, and cellular communication within the seminiferous epithelium. Consequently, the most effective interventions are likely to be those capable of simultaneously targeting multiple regulatory nodes rather than a single downstream pathway.

This perspective may also explain the variable efficacy observed among antioxidant therapies. Compounds that function exclusively as ROS scavengers may provide only partial protection if mitochondrial dysfunction, impaired autophagic flux, ferroptotic susceptibility, or inflammatory signalling remain unresolved. In contrast, interventions that restore redox balance while preserving physiological ROS signalling, mitochondrial integrity, and autophagic function are more likely to maintain cellular adaptation and prevent activation of RCD pathways. Future therapeutic approaches may therefore benefit from combining antioxidant, mitoprotective, autophagy‐modulating, and anti‐inflammatory strategies to enhance resilience of the seminiferous epithelium under thermal stress.

Taken together, current evidence supports a model in which antioxidant interventions act as modulators of an integrated redox‐dependent decision network rather than simple ROS scavengers. Their biological significance lies not only in reducing oxidative damage but also in preserving the regulatory systems that govern cell fate decisions. By simultaneously targeting mitochondrial dysfunction, autophagic imbalance, inflammatory signalling, and lipid peroxidation, these compounds promote cellular adaptation and maintain spermatogenic homeostasis under HS. Restoring the balance between ROS signalling, autophagic competence, mitochondrial function, and regulated cell death pathways may therefore represent the most promising strategy for preserving male fertility in an increasingly warming environment.

## VII. CONCLUSIONS

(1) Heat stress disrupts testicular homeostasis by altering redox balance and activating a coordinated network of cellular responses. Rather than acting solely as a source of oxidative damage, HS functions as a regulator of a redox‐dependent cell fate decision network that determines whether germ cells adapt, survive, or undergo regulated cell death.
(2) ROS occupy a central position within this network, functioning as quantitative signalling molecules that establish critical thresholds between adaptation and dysfunction. Consequently, the biological impact of HS depends not simply on ROS generation, but on the balance between ROS production and the capacity of antioxidant and proteostatic systems to preserve redox homeostasis.
(3) Autophagy functions as a molecular rheostat linking oxidative stress to cell fate decisions. Efficient autophagic flux preserves mitochondrial quality control, proteostasis, and cellular resilience, whereas autophagic dysfunction amplifies oxidative stress and promotes the transition from adaptation to regulated cell death pathways.
(4) Apoptosis, ferroptosis, and pyroptotic signalling represent interconnected execution programs that emerge when adaptive mechanisms fail to restore homeostasis. These pathways share common upstream triggers, including mitochondrial dysfunction, lipid peroxidation, autophagic failure, and persistent oxidative stress, highlighting their integration within a common redox‐dependent framework.
(5) The effects of HS vary according to the developmental stage and biological characteristics of individual cell populations. Spermatogonia exhibit relative resilience and preserve the stem cell pool, spermatocytes represent the principal vulnerability node due to their dependence on mitochondrial function and genomic integrity, post‐meiotic germ cells are particularly susceptible to defects in structural remodelling and membrane integrity, and Sertoli cells function as the central regulatory hub of the seminiferous microenvironment.
(6) Antioxidant interventions exert protective effects not only through attenuation of oxidative stress but also through preservation of mitochondrial function, autophagic competence, inflammatory control, and membrane integrity. Effective therapeutic approaches should therefore aim to restore the adaptive capacity of the redox‐dependent decision network rather than indiscriminately suppressing physiological ROS signalling.

## Data Availability

Data sharing not applicable to this article as no datasets were generated or analysed during the current study.
